# I. Foot-lameness. II. Fecal Stasis

**Published:** 1897-04

**Authors:** H. P. Miller

**Affiliations:** Student, Ohio State University Hospital


### REPORTS OF CASES.

### I. FOOT-LAMENESS. II. FECAL STASIS.

By H. P. Miller,
STUDENT, OHIO STATE UNIVERSITY HOSPITAL.

I. *Subject:* Bay draught-mare. *History:* Lameness developed suddenly while at work; grew steadily worse. *Symptoms:* Limb free from swelling or tenderness to the touch; animal reluctant to place weight upon the foot; pulsation in bloodvessels leading to the foot well marked. On removal of shoe and paring out of sole a large corn was disclosed.

II. *Subject:* St. Bernard dog. *History:* Persistent constipation for three weeks; belonging to a physician, a large number of cathartic preparations had been given, with no results. *Symptoms:* Animal emaciated and weak, a hard mass about the size of two fists could be felt in the region of the flank. *Treatment:* Warm-water injections administered per rectum through a rubber tube attached to a can, the latter placed at an elevation of ten feet. Continuous flow until twelve gallons were allowed to flow into the rectum, with manipulation externally of the mass; only a few fragments came away the first day. After four consecutive days' treatment by irrigation the mass disintegrated and was flushed out. Its removal was followed by a return of appetite and favorable symptoms leading to recovery promised.

